# Selection of Outcomes and Measurement Methods in Clinical Trials for Digital Therapeutics: Qualitative Analysis

**DOI:** 10.2196/86437

**Published:** 2026-10-15

**Authors:** Johannes Mahlmann, Anne Mainz, Vera Weirauch, Achim Mortsiefer, Sven Meister

**Affiliations:** 1 Health Care Informatics, Faculty of Health, School of Medicine, Witten/Herdecke University, Pferdebachstraße 11, Witten, 58448, Germany, 49 2302 92678608; 2 Department of Healthcare, Fraunhofer Institute for Software and Systems Engineering, Dortmund, Germany; 3 General Practice II and Patient-Centredness in Primary Care, Institute of General Practice and Primary Care, Faculty of Health, School of Medicine, Witten/Herdecke University, Witten, Germany

**Keywords:** digital therapeutics, DTx, digital health interventions, digital health evaluation, mobile health apps, qualitative research, core outcome set, outcome measurement, clinical efficacy trial, clinical trial methodology, measurement instruments

## Abstract

**Background:**

In recent years, the availability of digital therapeutic apps in medicine, digital therapeutics (DTx), has significantly increased. To evaluate the therapeutic efficacy of DTx, an increasing number of scientific studies have been conducted in this field. Several authors have pointed out that a standardized approach to DTx evaluation is relevant to improving the comparability of apps in the future. Currently, it is unclear how study authors proceed when selecting outcomes and measurement methods (O&M) for DTx studies.

**Objective:**

The primary aim of this study was to investigate how study authors currently select O&M in DTx studies, specifically for applications delivered in the form of apps. The secondary objectives were to collect suggestions for improving the comparability of DTx apps and studies in the future and to examine whether core outcome sets (COS) could represent a possible solution for enhancing comparability.

**Methods:**

This study was based on 19 semistructured interviews. All interview partners had prior experience in planning and conducting DTx studies. The interviews were conducted between August 2024 and November 2024. Subsequently, qualitative data analysis was performed according to Kuckartz and Rädiker using a deductive-inductive research approach.

**Results:**

The results of this study showed that the methodological approach for selecting O&M in DTx studies was primarily based on research, as well as on personal prior experience and individual ideas. Interview participants indicated that when selecting O&M, they particularly focused on aspects such as scientific quality criteria or practical implementation and feasibility. A suggestion for achieving better comparability of DTx studies in the future was to use standardized O&M per therapeutic indication area. Almost all interviewees considered COS to be a suitable approach for improving the comparability of DTx and associated studies. However, we also identified critical perspectives that questioned their application.

**Conclusions:**

Across professional groups, both commonalities and differences were observed in methodological approaches as well as in the selection of O&M in DTx studies. To improve the comparability of DTx interventions and trials, several approaches have been developed, for example, through the use of guidance frameworks, artificial intelligence programs, or the establishment of COS. However, further research is warranted to advance this field.

## Introduction

### Background

Digitalization has led to changes in health care at various levels [1]. Within this development, numerous terms have been established that describe the connection between digital innovations and health care. One example is the term digital therapeutics (DTx). According to the European Data Protection Supervisor [2], DTx are defined as “evidence-based therapeutic interventions driven by software to prevent, manage, or treat a medical disorder or disease. In other words, DTx are patient-facing software applications that help patients treat, prevent, or manage a disease and that have a proven clinical benefit” [2]. Thus, DTx (1) are software-based apps; (2) are intended primarily for patients rather than health care providers or third parties; (3) can be distinguished according to their primary therapeutic objective into apps intended for disease treatment, disease management, or the improvement and/or prevention of health care functions; (4) are based on concrete therapeutic concepts; and (5) have demonstrated their therapeutic efficacy through scientific studies [2-8].

In the European context, DTx are classified as medical devices and consequently assigned to risk classes I-III [9]. They are therefore subject to authorization requirements comparable to those for other medical devices or pharmaceuticals [10].

Possible technical realizations of DTx include web apps, smartphone apps, or serious games [4,11]. Currently, numerous apps are available for various medical conditions, including mental disorders, cardiovascular diseases, addiction disorders, cancer, and pain therapy [11,12]. DTx must be clearly distinguished from fitness and lifestyle apps that do not meet DTx criteria and whose efficacy has not been demonstrated in clinical studies [6,13,14].

Typically, multiple studies are carried out throughout the development and authorization of DTx. Study objectives and the selection of outcomes and measurement methods (O&M) usually differ depending on the technical development phase and scientific question. Possible research questions may include user experience, usage duration, or treatment cost reduction of an app [15-18]. As previously mentioned, one quality characteristic of DTx is that therapeutic efficacy must be demonstrated in studies [14,19]. Internationally, there is currently a lack of uniform requirements regarding what should constitute efficacy evidence and which criteria must be fulfilled for app authorization [6,9,12,20,21].

Germany has developed a structured authorization and reimbursement process for DTx through the Digital Healthcare Act and the fast-track procedure for digital health applications (DiGA). The German procedure includes requirements for data protection, user-friendliness, and demonstration of positive care effects. Study end points for efficacy demonstration may include health status, illness duration, survival, morbidity, mortality, or quality of life, with the latter being particularly patient relevant [22]. With reference to this system, several countries have taken the German system as a model and have initiated the development of comparable frameworks [23,24]. For Europe, France with the Prise en Charge Anticipée model and Belgium with the National Institute for Health and Disability Insurance (INAMI-RIZIV) model are particularly noteworthy, both offering concrete requirements for authorization processes and app reimbursement [20].

The definition of a high-quality DTx depends on the evaluation criteria applied by different stakeholders, including patients, clinicians, health care payers, developers, and policymakers, whose perspectives and priorities shape their individual assessments [25]. Therefore, assessments of DTx quality vary substantially across contexts and stakeholder interests. The Digital Therapeutics Alliance (DTA), which is one of the leading international nonprofit organizations dedicated to the research and development of DTx, has emphasized that there is currently no unified, internationally accepted assessment framework that is independent of individual stakeholder interests [25]. To promote harmonization in the evaluation of DTx, the DTA published the Value Assessment and Integration Guide [25]. This guide provides a structured approach for the holistic assessment of DTx, independent of therapeutic area and evaluating stakeholder group.

A complementary approach is the DTx Real-World Evidence Framework developed by Kim et al [26], which offers a scientifically grounded and clearly structured methodology for the design, development, evaluation, and long-term monitoring of DTx. The authors demonstrate the application of this framework using a concrete example, placing particular emphasis on the design of DTx studies under real-world conditions and on key considerations in the selection of appropriate comparator groups [26].

Taken together, these approaches provide valuable guidance on how DTx can be developed and evaluated following implementation. However, these frameworks offer limited actionable guidance regarding the design of individual DTx studies and the key considerations required to enable long-term comparability of DTx based on study results.

As noted by various researchers, inconsistent or a lack of guidelines specifically addressing the selection of O&M in DTx studies can lead to methodological problems [17,27-29]. While individual app efficacy can be demonstrated through scientific studies, inconsistent O&M across different studies severely limits comparability between studies and DTx, assuming that apps within the same treatment area should be compared [17,27-29]. Given the predicted further increase in DTx numbers and associated studies, comparability is fundamentally important for making evidence-based therapeutic decisions and answering reimbursement questions [30,31]. The use of core outcome sets (COS) has been mentioned in the literature as a potential solution for achieving a minimum level of comparability and study quality in DTx studies but has not been empirically evaluated yet [17,28].

COS are defined as “sets [that] represent the minimum that should be measured and reported in all clinical trials of a specific condition [...] The existence or use of a core outcome set does not imply that outcomes in a particular study should be restricted to those in the relevant core outcome set. Rather, there is an expectation that the core outcomes will be collected and reported, making it easier for the results of studies to be compared, contrasted and combined as appropriate; while researchers continue to explore other outcomes as well*”* [32].

### Objectives

Although the problem of poor comparability between DTx studies and apps due to inconsistent O&M has been repeatedly noted in the literature, it remains unclear how O&M are currently selected in DTx studies. In our view, addressing these issues requires 2 key steps. First, current practices among study authors need to be examined to understand the criteria they consider when selecting O&M for DTx studies. Second, study authors with prior experience in DTx research should be asked to assess whether they consider COS to be a suitable solution or to propose alternative approaches. On the basis of this literature-derived rationale, the following 3 research objectives were developed for this study:

1. To determine how O&M are currently selected in DTx studies specifically for DTx in the form of apps
2. To collect ideas for achieving better comparability of DTx studies and apps in the future
3. To obtain an assessment of whether COS could contribute to improving comparability of DTx studies and apps

## Methods

### Overview

Qualitative research methods are established approaches in the context of medical research and health services research. They are particularly suitable for gaining a better understanding of individuals’ decisions and thought processes. Within qualitative research, different methodological traditions and schools of thought exist. For this study, we chose the approach by Kuckartz and Rädiker as the theoretical foundation. Following this approach, qualitative content analysis was applied as the methodological procedure, as it is regarded as one of the most suitable qualitative methodologies for the research aims in the context of digital health research and was the approach with which the research team had the greatest prior experience [33]. The presentation of the study findings is guided by established qualitative research practices and recommendations and combines an objective, partly quantitative presentation with a complementary interpretive perspective on the interviewees’ statements [34-37]. To ensure methodological rigor, this study follows the COREQ (Consolidated Criteria for Reporting Qualitative Research) checklist (Checklist 1) [38].

### Sampling and Recruitment

To obtain answers to the research questions from various perspectives, we decided on purposeful sampling for recruiting interview participants until thematic saturation was reached. Inclusion criteria were participants who (1) could demonstrate topic-related research activity, (2) had published topic-related publications, (3) could demonstrate topic-related professional activity (eg, health insurance or development, distribution, or application of medical apps or DiGA), or (4) were involved in creating legal guidelines for DiGA.

These inclusion criteria were chosen in line with the overarching aim of our research project, which sought to conduct an in-depth analysis of the planning and implementation of DTx studies and to develop recommendations for improving their comparability. In our view, prior experience in this field was necessary to adequately address these research questions. Stakeholder groups, such as end users of DTx or physicians who prescribe these apps, would not have been able to address the specific research questions of this study in the same depth, as they are not directly involved in study design and implementation. We therefore excluded these groups from participation, as they were not expected to provide the insight required for this investigation. On the basis of these criteria, we contacted individuals and companies through national networks and the DiGA directory. The DiGA directory is a German BfArM (Bundesinstitut für Arzneimittel und Medizinprodukte) registry that lists manufacturers who have submitted DiGA applications, shows which applications are under review or approved, and provides patients and physicians with an overview of prescribable apps for various medical conditions [39]. When we became aware of specific DTx studies through these sources, we also contacted the study authors.

### Data Collection

Problem-centered interviews according to Witzel and Reiter [40] were conducted between August 2024 and November 2024 via videoconferencing programs. At the request of participants, 2 interviews were carried out via telephone due to organizational considerations. All interviews were facilitated by a single researcher (JM) who had received formal training in qualitative interviewing.

To prepare for the interviews, we developed a semistructured interview guide (Multimedia Appendix 1). The guide’s development was informed by the publications discussed in the *Introduction* section, which highlight that inconsistent or a lack of guidelines for the selection of O&M in DTx studies can lead to methodological challenges. The guide was pretested and collaboratively revised with 4 individuals (A Mainz, VW, SM, and A Mortsiefer). All contributors had several years of professional experience as well as expertise in research on and with DiGA. The pretest supported the structuring of the interview guide into clear thematic sections and helped refine it to focus on questions relevant to the research objectives.

At the beginning of each interview, the interviewer briefly introduced himself and explained the study using an example. Interview partners were informed that this study aimed to investigate how O&M are selected in clinical studies of DTx delivered in the form of mobile apps. The subsequent interview guide could be divided into 4 content sections. The first section asked interview partners about personal characteristics (eg, age, current professional activity, and participation in DTx studies). The second interview section focused on methodological approaches and content aspects in O&M selection for DTx studies. Questions about the methodological approach aimed to understand how O&M are selected in DTx studies (eg, literature review or application oriented). Questions about content aspects addressed what they considered when selecting O&M (eg, financial aspects or clinical relevance). In the third interview section, participants were presented with statements from other authors emphasizing that, at present, meaningful comparisons across different DTx apps and across different DTx studies remain largely infeasible, as studies use heterogeneous O&M. Participants were then asked about possibilities they saw for achieving better comparability of DTx apps and studies in the future. In the fourth interview section, participants were introduced to COS as a possible solution for improving comparability and study quality based on the definition provided in the *Introduction* section and by Webbe et al [41]. Following this definition, interviewees were asked for their opinions on having COS specifically for DTx within each therapeutic area, such as depression therapy or pain therapy. After completing all interview questions, interview partners had the opportunity to express themselves on topic-related but independent matters and provide suggestions or feedback.

### Data Analysis

After recording, interviews were imported into MAXQDA 2022 software (VERBI Software GmbH), automatically transcribed, and subsequently checked by 1 person (JM) for completeness, transcription accuracy, and depersonalization. Following this process, the transcripts were grouped in the MAXQDA software program according to the professional background of the interviewees to enable a comparative analysis of methodological approaches and substantive considerations in the selection of O&M across professional groups. Subsequent content processing and analysis were conducted manually in accordance with the qualitative content analysis approach of Kuckartz and Rädiker [42-44]. Methodologically, the study follows a deductive-inductive procedure that combines thematic structuring and evaluative elements, supported by thematic statements and complemented by a quantitative representation where appropriate.

The primary (deductive) coding system was developed based on the semistructured interview guide. This system was tested on 6 interviews and reevaluated by the research team (JM, VW, SM, and A Mainz). The primary coding system can be found in Multimedia Appendix 2. To develop the secondary (inductive) coding system, all interviews were first coded with the primary coding system. Brief summaries were then written for all coded text segments, and subcodes were subsequently derived from these summaries. This methodological approach was guided by the recommendations of Kuckartz and Rädiker [42,44] regarding the development of primary and secondary coding systems for qualitative research. After developing the secondary coding system, it was reevaluated by the research team (JM, SM, and VW), leading to the addition of the subcode category “Requirements for COS.” The secondary coding system, including definitions and text examples, can be found in Multimedia Appendix 3. To validate the completeness of the coding framework, all interview texts were reread completely by JM to identify any additional segments that could either be assigned to the existing coding system or would require the development of an additional code category. At this stage, we observed that all relevant statements made by interview participants could be assigned to existing code categories and that the categories showed sufficient conceptual distinction for the planned analysis and presentation of results. In accordance with the recommendations of Kuckartz and Rädiker [42-44], this was considered the point of thematic saturation. Although saturation was reached after 15 interviews, additional interview appointments had already been scheduled; therefore, these were conducted and included in the analysis.

For the presentation of our research findings in this publication, all interview quotations from German-speaking participants were translated into written English.

### Ethical Considerations

The study procedure and the interview guide were reviewed by the Ethics Committee of Witten/Herdecke University and granted positive ethical approval on May 23, 2024 (application S-120/2024).

Prior to participation, all potential interview participants received an information package describing the aims and procedures of the study, together with a data protection information sheet and a written informed consent form. Interviews were conducted only after participants had provided written informed consent. Immediately before the interview began, participants were reminded that their participation was voluntary and that they could withdraw from the study at any time without providing a reason.

To protect participants’ privacy and confidentiality, all collected data were deidentified prior to analysis. As part of the deidentification process, interviews were assigned sequential identification numbers according to the order in which they were conducted. All interview transcripts were carefully reviewed to remove or redact any information that could potentially identify the participants or reveal their place or position of employment.

Participation in this research project was entirely voluntary. Participants did not receive any financial compensation or other form of remuneration for their participation.

## Results

### Characterization of Interview Partners and Interviews

This qualitative research project interviewed 19 participants (men: n=12; women: n=7) based on a semistructured interview guide. For the analysis of the collected data, participants were grouped by professional role as follows: 7 researchers, 6 physicians and psychologists, and 6 entrepreneurs and consultants. Participants’ ages ranged from 27 to 72 (mean 43, SD 11; range 27-72) years. On average, participants reported 17 years of professional experience and had conducted 7 DTx studies. The number of DTx studies conducted in app form ranged from 1 to 20 (median 5, IQR 4-8.5) studies. Of the 19 interview partners, 16 indicated having primarily experience conducting DTx studies in Germany. An overview of this information can be found in Multimedia Appendix 4.

The average interview duration was 29 minutes, with the shortest interview lasting 10 minutes and the longest lasting 50 minutes (median 25 min).

### General Results

The analysis indicated that thinking and decision-making in the planning and conduct of DTx studies were grounded in a range of methodological approaches and shaped by differing evaluations of key conceptual aspects. Reflecting both convergent and divergent approaches to the design and implementation of DTx studies, participants also articulated diverse perspectives on how DTx research might be further advanced and refined in the future.

Figure 1 provides an overview of all interviews as well as the codes and subcodes identified within each interview section during the qualitative content analysis. For completeness and ease of reference, the corresponding data are also presented in tabular format in Multimedia Appendix 5.

According to Kuckartz and Rädiker [42], quantitative analyses within qualitative research projects represent a valuable means of obtaining an initial overview of qualitative data. They help identify thematic emphases as well as similarities and differences within the dataset, thereby providing the basis for the subsequent qualitative interpretation. However, no conclusions regarding the substantive significance or relative importance of the identified themes can be drawn from this quantitative overview. Such interpretations are therefore reserved for the subsequent qualitative analysis of the findings.

The figure is structured as follows. The top row lists the professional groups to which the interview participants were assigned based on their self-reported professional background. The second row contains the interview identification numbers, which were assigned according to the chronological order in which the interviews were conducted. The gray-shaded rows with larger font (eg, Methods, Requirements, or Core outcome sets) indicate the main thematic sections of the interview guide and serve to structure the figure conceptually.

The left-hand column lists the assigned codes together with their corresponding subcodes. Bold type indicates the higher-level codes within each interview section. For example, the category Methods>Measurement methods refers to the interview section in which the methodological approach to the selection of O&M is discussed, with the coded statements relating specifically to measurement methods. The left-aligned, nonbold entries listed beneath represent the respective subcodes within this coding category. The final column on the right-hand side of the figure indicates the number of interviews in which the respective subcode was assigned at least once.

**Figure 1.**
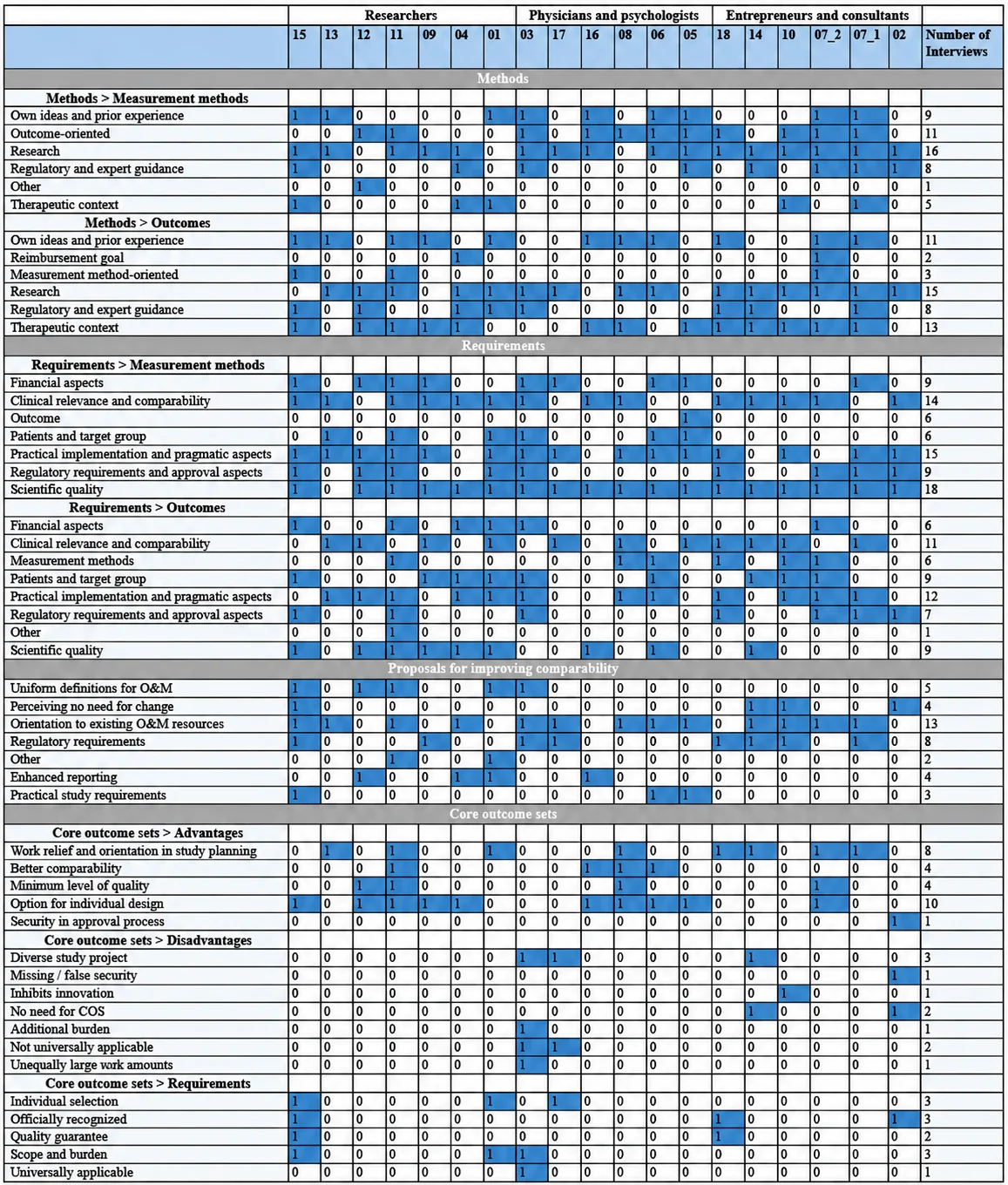
Distribution of subcode categories across interview groups. COS: core outcome set; DTx: digital therapeutics; O&M: outcomes and measurement methods.

### Descriptive Analysis of the Code Distributions

The first section of Figure 1 relates to the interview questions addressing the methodological approach to selecting O&M in DTx studies. The interview data were analyzed separately depending on whether participants referred to the selection of Measurement methods or Outcomes.

For both the selection of Measurement methods and Outcomes, the subcode category Research was the most frequently assigned category. It was coded in 16 of the 19 interviews in relation to the selection of Measurement methods and in 15 interviews in relation to the selection of Outcomes. Thus, this category was represented in almost all interviews and across all professional groups.

In contrast, marked differences between the selection of Measurement methods and Outcomes were observed for the subcode category Therapeutic context. While this category was assigned only five times in relation to the selection of Measurement methods, it was identified in 13 interviews concerning the selection of Outcomes.

A similar pattern emerged for the subcode category Outcome-oriented, which was coded in 11 interviews addressing the selection of Measurement methods, whereas the corresponding counterpart, Measurement method-oriented, was identified in only 3 interviews concerning the selection of Outcomes. Overall, participants more frequently described the selection of measurement methods as being guided by predefined outcomes than the selection of outcomes as being determined by already established measurement methods.

Differences were also observed regarding the thematic breadth of the interviews. For example, interviews 15 and 07_01 covered a wide range of different subcode categories within this topic area, whereas interviews 02, 09, and 17 contained comparatively few distinct subcodes related to the methodological approach.

The second section of Figure 1 summarizes the requirements considered by the interview participants when selecting O&M.

Regarding Measurement methods, the subcode category Scientific quality was the most frequently represented category, appearing in 18 of the 19 interviews. This was followed by Practical implementation and pragmatic aspects (15 interviews) and Clinical relevance and comparability (14 interviews).

A different distribution pattern emerged for the requirements relating to Outcomes. Here, Practical implementation and pragmatic aspects was the most frequently assigned subcode category, occurring in 12 interviews. This was followed by Clinical relevance and comparability (11 interviews) and the subcode categories Scientific quality and Patients and target group, each of which was represented in 9 interviews.

Differences between professional groups were particularly evident for the subcode category Scientific quality. Scientific quality was represented in 18 of the 19 interviews addressing the requirements for Measurement methods, indicating that this aspect was consistently considered across the dataset. In contrast, in discussions of the requirements for Outcome selection, Scientific quality was assigned predominantly in interviews with researchers, whereas it was represented considerably less frequently in interviews with physicians and psychologists as well as entrepreneurs and consultants.

Within the professional group of entrepreneurs and consultants, 2 notable patterns emerged. First, the subcode category Financial aspects was assigned only once in relation to the requirements for Measurement methods within this professional group. Second, the subcode category Regulatory requirements and approval aspects was assigned more frequently in relation to the requirements for Outcomes than in the other professional groups. Both patterns differed from the remaining professional groups, in which Regulatory requirements and approval aspects were discussed less frequently and Financial aspects more frequently.

The third section of Figure 1 addresses proposals for improving the comparability of DTx studies and apps. The most frequently represented subcode category within this interview section was Orientation to existing O&M resources. Statements assigned to this code described the use of existing O&M resources, such as guidance systems or standardized O&M approaches for specific indications, as an appropriate strategy to improve the comparability of studies and apps. This category was represented across all professional groups and in 13 of the 19 interviews.

Professional group–specific differences were particularly evident for the subcode categories Uniform definitions for O&M and Regulatory requirements. Statements referring to Uniform definitions for O&M occurred more often in interviews with researchers, whereas Regulatory requirements were more frequently assigned in interviews with entrepreneurs and consultants.

The final section of Figure 1 summarizes participants’ views on whether COS represent a suitable approach for improving the comparability and quality of DTx studies and apps.

Among the perceived advantages of COS, the subcode categories Option for individual design (10 interviews) and Work relief and orientation in study planning (8 interviews) were assigned most frequently. Overall, advantages were discussed more often than potential disadvantages (27 vs 11 coded segments).

The distribution of the codes also revealed distinct response patterns across interviews. In 11 of the 19 interviews, participants discussed only the advantages of COS without simultaneously mentioning potential disadvantages or requirements for their development.

### Selection of O&M in DTx Studies

#### Overview

The subsequent sections of the results present the qualitative findings of this study. The second part of the interviews addressed the selection of O&M in DTx studies. Participants were asked about both their methodological approach to selecting O&M (ie, how they proceed when selecting O&M for a DTx study) and the content-related aspects considered during this process (ie, which criteria they consider when selecting O&M).

#### Research as a Foundation

Many interviewees described during the conversations that they were not aware of any universal master plan for how DTx studies should be methodologically designed. Rather, each study was understood as an individual project that required fundamental reconceptualization from the outset. Given the diversity of thematic and therapeutic approaches across different DTx apps, the initial phase of a study project was described as requiring basic orientation.

Across all professional groups, participants reported conducting a search activity as a central methodological component of this process. This activity was described as supporting the identification of O&M that might be suitable for scientifically evaluating the respective app. This practice was also reflected in the data presented in the Methods section of Figure 1, where decisions regarding outcome selection and measurement methods were most frequently grounded in prior research.

The scope and structure of the search process described by interviewees varied considerably. While some participants reported conducting rather informal and unsystematic searches, others described carrying out more structured searches in the form of systematic literature reviews in accordance with scientific quality criteria:

*Well, I would specifically conduct a literature review to see which clinically validated, or clinically tested tools are available for the disease entity or application*.

[Interview 09, researcher]

#### Drawing on Established Experience

Beyond research-based approaches, interviewees reported that the selection of O&M for DTx studies was also informed by prior professional experience. The professional background of participants appeared to shape the type and relevance of experience drawn upon in this process.

Researchers predominantly referred to methodological experience, particularly regarding test procedures or study designs that they or others had previously applied in comparable research contexts:

*We use proven methodologies that we have often applied and that are well known*.

[Interview 13, researcher]

In contrast, physicians and psychologists emphasized the integration of clinical expertise, particularly their knowledge of specific disease patterns and practical experience working with patients:

*I actually often use tests from my clinical experience that have also proven to be relevant and effective in clinical studies*.

[Interview 16, physician and psychologist]

Similarly, entrepreneurs and consultants drew on regulatory knowledge and insights from approval processes. They also considered design strategies observed in competing DTx products with comparable therapeutic indications. In doing so, they took into account how similar apps had structured their studies, which methodological choices had been accepted in previous approval processes, and which approaches appeared to be most successful in terms of market authorization and positioning.

The substantive value of prior experience, as well as the degree of systematicity with which it was incorporated into the planning process of DTx studies, can be illustrated by the following finding. A considerable number of interviewees described developing personal databases based on accumulated experience, a pattern that was also reflected in the coded data. During the planning process, particularly when selecting O&M, these databases enabled them to draw systematically on previously acquired knowledge and experiences to guide their decision-making:

*I have gradually created a sort of database in which I collect well-known measurement instruments. Their advantages and disadvantages are listed, so that I can already make a selection to some extent*.

[Interview 06, physician and psychologist]

Efficiency in planning and conducting DTx studies was closely linked to both the breadth of prior experience and the degree to which this experience was systematically documented, indicating that experience functions not only as accumulated knowledge but also as an operational resource in study design.

Across groups, prior experience thus functions as a pragmatic heuristic that reduces complexity and compensates for uncertainty in study planning, albeit with different profession-specific emphases.

#### External Stakeholder Input and Validation

Across all professional groups, a recurring practice in the selection of O&M for DTx studies was the early exchange with external stakeholders. These included clinical experts, methodological specialists in DTx research, and regulatory authorities.

These exchanges were primarily used to discuss and validate planned study designs, particularly with regard to the appropriateness of selected end points and measurement procedures. The overarching aim was to ensure that the chosen measures were both scientifically suitable for evaluating the respective DTx app and aligned with expectations relevant for later approval and evidence generation. In this sense, external input functioned as an early orientation and validation mechanism during the study design phase.

As most interviewees had experience with the German regulatory context, particularly the BfArM framework, interactions with regulatory authorities were often described as taking place within a relatively structured approval environment. However, similar patterns were also reported in other contexts, including the United States:

*So right now, with the study in the United States, we met with the FDA very often before—although they didn’t really have a proper procedure yet, at least not like in Germany, but something similar. And of course, we talked a lot about it. What is the endpoint? What should the comparison group look like? Should the primary endpoint be after three or after six months? What is the minimal clinically important difference? Where does it come from? How do we use it to classify the difference between groups? Do we dichotomize it, or do we look at the group mean and that?*

[Interview 15, researcher]

### Key Considerations Guiding the Selection of O&M in DTx Studies

Analysis of content criteria considered when selecting O&M in DTx studies revealed a differentiated picture of various priorities and requirements, partially differing between professional groups. As shown in the Requirements section of Figure 1, interviewees particularly considered aspects related to clinical relevance and comparability, practical implementation and pragmatic aspects, as well as scientific quality when selecting O&M.

#### Scientific Rigor

In the selection of measurement methods, scientific quality criteria emerged as a central aspect across all groups of interviewees. Participants emphasized psychometric properties, including validation of O&M for specific target populations, reliability, and indicators such as the minimal clinically important difference (MCID).

The MCID addresses a persistent challenge in medical research and patient care, namely that objectively measured parameters may show statistically significant changes during treatment, while these changes are not necessarily perceived as meaningful by patients themselves. To address this discrepancy, the development and application of MCIDs have gained increasing importance. For specific diagnostic measures or outcomes, the MCID defines the smallest change in score that is considered clinically meaningful for an individual patient [45]. In the context of planning and conducting DTx studies, MCIDs were described as particularly relevant for assessing whether a DTx app provides a meaningful therapeutic benefit for patients.

Questionnaires were reported as one of the most frequently used methods for evaluating DTx interventions. In this context, interviewees highlighted the importance of linguistic adaptation and validation across different populations. Beyond language, factors such as age-specific suitability and disease-specific relevance were described as requirements that must be covered by prior validation of the instruments before selection.

Regarding the selection of appropriate measurement instruments, interviewees highlighted the challenge of identifying tools that adequately meet the methodological and clinical requirements of the respective study:

*You need to choose a sufficiently valid instrument that can measure the intended endpoints. This is often challenging, because not all instruments have all the necessary psychometric properties*.

[Interview 03, physician and psychologist]

#### Pragmatic Implementation

Beyond scientific considerations, a substantial number of statements emphasized the importance of practical constraints in the implementation of DTx study designs in real-world clinical settings. Across interviews, study planning was consistently shaped by the feasibility of integrating methodological approaches into hospital or therapeutic environments. Available resources, such as equipment or tools, functioned as key decision criteria by enabling or constraining the selection of specific O&M:

*It should be a measurement method that is simple to perform, where perhaps the necessary measuring devices are already available*.

[Interview 13, researcher]

A further recurring theme concerned the burden associated with study implementation for both study teams and patients. More complex study designs were described as requiring additional personnel resources or specifically trained staff, which were not always available in sufficient capacity. For patients, minimizing study-related burden was repeatedly highlighted as essential to reduce dropout and ensure continued participation throughout the study period:

*So when do we assume that we can detect a change, because we actually want to measure as early and infrequently as possible to avoid burdening patients and have the lowest possible dropout*.

[Interview 01, researcher]

In addition, timing considerations for outcome assessment were described as relevant not only in relation to feasibility and burden but also in response to regulatory requirements. Regulatory time frames were reported to influence study planning, particularly regarding the expected time frame in which clinical improvement must be demonstrated. Consequently, measurement approaches requiring longer observation periods were considered unsuitable under such constraints.

#### Clinical Applicability and Relevance

Moreover, a recurring consideration in the selection of O&M was the preference for those that had already demonstrated relevance in both clinical practice and scientific research.

From a medical perspective, interviewees emphasized the importance of selecting end points that are routinely used in clinical practice. This was described as facilitating a more accurate assessment by health care professionals of whether, and to what extent, a DTx app is effective in the treatment of a specific condition and what therapeutic benefit it may provide to patients. One interviewee from the group of physicians and psychologists (interview 05) particularly highlighted that, from a clinical standpoint, hard end points—such as absolute weight reduction, improvements in joint range of motion, or prolonged survival—were considered more relevant outcome measures. In contrast, soft outcomes, such as general well-being, were described as less suitable for demonstrating the clinical effectiveness of a DTx intervention:

*So that was one of the decisive criteria for me, that it is a well-studied parameter where I can really say it is also significant for patient prognosis. So not just a parameter that might change positively, but it was important for me that this is a marker where you can really say this is significant for this patient group when it improves.*

[Interview 05, physician and psychologist]

From a scientific perspective, comparability across DTx and related research projects was closely linked to the use of identical or well-established O&M within a given context. Established measurement approaches were also associated with higher acceptance of study findings within the scientific community, including their likelihood of publication.

### Perspectives on Improving the Comparability of DTx Studies

#### Overview

The focus of the third section of the interviews was on identifying approaches that could improve the comparability of DTx apps and the associated clinical studies. The qualitative analysis revealed 3 broad perspectives. First, participants proposed strategic measures aimed at establishing common methodological standards. Second, several interviewees described concrete technological solution concepts that could support greater comparability in practice. Finally, a minority of participants questioned whether additional standardization was necessary at all. An overview of the identified approaches can be derived from the corresponding subcode categories presented in the Proposals for Improving Comparability section of Figure 1.

#### Building Comparability Through Guidance and Regulation

The primary and most frequently discussed recommendation focused on the development of guidance systems and standardized O&M tailored to specific clinical indications (Figure 1). Participants largely agreed that a pragmatic way to improve comparability would be to define a clear framework through which appropriate and unified O&M could be identified for individual research projects. This approach was described as relying on expert-driven curation to establish a baseline set of outcomes or measurement tools—potentially including 5 to 15 key markers—for specific therapeutic areas, such as depression or diabetes management.

While some respondents highlighted medical professional societies as a suitable body to organize and oversee these standards, the data suggest a broader range of possible implementation pathways. The main objective remained the establishment of broad consensus on which parameters are most relevant for a given disease, regardless of whether this consensus is led by professional societies, multistakeholder initiatives, or regulatory bodies:

*So I think it’s important to first agree on, at a high level, which parameters are actually important for a given disease and should be investigated. Then, for each condition, there could be a handful— maybe five to ten or even up to fifteen—markers that play a role*.

[Interview 08, physician and psychologist]

*But maybe professional societies should handle this, to say, okay, in the case of back pain, no matter who publishes what [no matter who conducts a DTx study], when it comes to tackling back pain, these are the required measurement tools, so that we can actually compare results. I mean, that would be the simplest approach*.

[Interview 06, physician and psychologist]

Recognizing that voluntary and consensus-based guidelines may not be sufficient to ensure broad implementation, a second thematic cluster emphasized the need for formal legal and regulatory requirements (Figure 1). This recommendation shifts the discussion from optional best practices toward binding requirements directly linked to market approval and reimbursement pathways.

Within this perspective, national regulatory authorities were described as important gatekeepers that could limit the range of acceptable methodological tools. Rather than leaving the selection of questionnaires entirely to study authors, regulatory bodies could define binding frameworks of approved diagnostic instruments:

*It is then the task of the regulatory authorities to provide guidance so that potential comparability can be achieved. [...] they [the regulatory authorities] should say,* “*Okay, we accept these and those questionnaires.” Everything else can be disregarded*.

[Interview 18, entrepreneur and consultant]

This call for regulatory intervention is further nuanced by an international dimension. Respondents highlighted that national legislative changes do not occur in isolation. Rather, they are increasingly influenced by emerging international evaluation frameworks. To prevent fragmented, conflicting national requirements that would complicate cross-border data comparison and integration, participants emphasized that future legal standardization must be harmonized internationally to ensure long-term, macroeconomic comparability (interview 03).

#### Architectural Archetypes for Practical Solution Concepts

Beyond these broad strategic recommendations, several participants proposed concrete technological and infrastructural solutions that could support greater comparability in practice. Although differing in complexity and scope, these concepts can be grouped into 3 architectural archetypes according to their primary mechanism, target audience, and level of systemic intervention (Table 1).

**Table 1.** Architectural archetypes for practical solution concepts.

Solution archetype	Primary mechanism	Target audience	Systemic intervention level
Information layer (eg*,* traffic light systems*)*	Visual reduction of multidimensional clinical data into simplified, discrete heuristic signals	Clinicians, end users, and nonexpert stakeholders	Low or downstream leaves existing trial designs untouched; standardizes output presentation.
Methodological layer (eg, knowledge repositories and GenAI^a^)	Semantic aggregation of historical trial data into active design “cookbooks” and conversational AI assistants	Study authors, R&D^b^ teams, and clinical researchers	Medium or midstream standardizes researcher behavior through historical benchmarking.
Structural layer (eg, centralized platform trials)	Complete systemic institutionalization of a continuous, multiarm clinical environment using a shared control architecture	Regulatory bodies, health insurers, and all DTx^c^ vendors	High or upstream eliminates decentralized trial autonomy; standardizes the environment.

a GenAI: generative AI.

b R&D: research and development.

c DTx: digital therapeutics.

The information layer represents an approach to post hoc visual simplification. Rather than standardizing how trials are conducted, this approach aims to standardize how results are communicated. The suggested mechanism was a simplified visual heuristic, specifically a “traffic light system” (interview 01). This solution avoids the complex task of mathematically aligning heterogeneous effect sizes across different study designs. Instead, it introduces a simplified binary or ternary evaluation filter (eg, green=proven effect on the primary end point). Its main value lies in supporting nonexpert decision-makers, such as patients and prescribing physicians, by reducing clinical complexity and enabling faster comparisons of app effectiveness.

The second archetype, the methodological layer, focuses on knowledge augmentation and AI-guided support during the study design phase, aiming to reduce future study fragmentation by directing researchers toward established best practices. This category reflects a technological development that begins with static data aggregation and evolves into more dynamic, intelligent support systems. At its foundation is a knowledge repository, often described by participants as a “cookbook,” which collects data from successful historical DTx approvals. By summarizing the most relevant outcomes, measurement instruments, and target populations for specific indications, such a registry could serve as a structured reference point for future study designs and reduce arbitrary variation (interview 07_01).

Building on this data foundation, the concept can evolve from a passive repository into an active generative support system. Through the integration of conversational AI, the effort required for literature reviews and study planning could be significantly reduced. This more advanced version would allow researchers to search historical trial designs and receive synthesized recommendations for their own clinical context:

*I just had the idea for something like a ChatGPT for studies, study results and study designs. So basically what we do manually, that you can type or speak somewhere and then get an overview of studies in the area of, for example, ophthalmology, eye diseases.*

[Interview 07_02, entrepreneur and consultant]

The third archetype, the structural layer, proposes institutionalized platform trials as a solution targeting the broader infrastructure of clinical evidence generation. Instead of harmonizing decentralized trials after they have been completed, this concept proposes a centralized research infrastructure in the form of publicly or insurer-funded platform trials:

*That’s a continuously running clinical study where new arms can be added and ended again. You need infrastructure for that. [...] insurance companies in Germany, which ultimately have to pay for the applications, conduct such a permanent trial. Whenever someone has a new app, it has to go in there*.

[Interview 15, researcher]

The central innovation of this archetype lies in integrating all trial variables within one permanent institutional framework. By evaluating competing DTx apps within the same study environment, each app could be assessed against the same control group, with identical outcome measures and within the same target population. In this way, the problem of comparability could be addressed directly by replacing fragmented, independent vendor-led trials with a unified research structure.

#### Counterperspective—Perceiving No Need for Change

Although most participants proposed measures to improve comparability, the qualitative analysis also revealed a counterperspective that questioned the need for standardization. A minority of participants explicitly defended the status quo (“Perceiving no need for change”; Figure 1), describing methodological heterogeneity not as a problem but as a natural feature of scientific practice and innovation. Drawing comparisons to the pharmaceutical sector, these respondents argued that drug approval processes also function without strict cross-trial standardization of measurement tools. From this perspective, introducing COS into the DTx field would be unnecessary and potentially constitute counterproductive regulatory overreach:

*Well, I find that studies for different products are not completely comparable, I have no problem with that at all. That is scientific life, scientific practice for me*.

[Interview 02, entrepreneur and consultant]

*Especially since I, as already outlined, rather question whether this is needed at all. This doesn’t exist in the pharmaceutical sector in that sense either*.

[Interview 14, entrepreneur and consultant]

### COS as a Promising but Complex Solution

The final section of the interviews addressed whether COS represents a suitable instrument for improving the comparability of DTx studies and apps. The statements of the interviewees revealed a highly differentiated picture. While participants generally recognized the great potential of COS to standardize and improve clinical research, they also emphasized significant practical challenges, potential risks for trial design, and a critical lack of formal regulatory backing.

The primary benefit associated with the integration of COS into the DTx ecosystem was the systematic facilitation of the study planning process. For research teams, selecting appropriate, well-established outcomes is often a complex and time-consuming task. A predefined COS serves as a practical orientation guide that reduces the initial effort required when designing a new study. By providing a clear framework of established parameters, it directly helps study teams optimize their workflow and ensures that critical clinical dimensions are not overlooked during the conceptualization phase:

*If you strive to do qualitatively good research, then you will gladly accept having to meet certain criteria and might even be happy to get guidelines where you can orient yourself and also forget less*.

[Interview 08, physician and psychologist]

Beyond lowering the organizational workload for research teams, the widespread adoption of COS was expected to have a strong stabilizing effect on the overall quality of DTx evaluations. When individual studies consistently align with relevant core outcomes, the resulting data automatically become more homogeneous. This structural consistency directly contributes to the macro-level objective of ensuring comparability across study results from different DTx studies.

Importantly, supporters of this approach pointed out that using a COS does not mean sacrificing scientific freedom or product-specific nuances. Because a COS is conceptually defined as a minimum baseline, it explicitly allows for a flexible, 2-layered study architecture. Researchers can easily fulfill the requirements of universal comparability while maintaining the freedom to include additional, customized parameters to capture the unique features of their specific digital app:

*You have a framework that ensures comparability and simultaneously freedom to include your own parameters*.

[Interview 16, physician and psychologist]

Despite these apparent benefits, the qualitative analysis revealed serious counternarratives that questioned the methodological consequences and the entrepreneurial viability of COS.

The first major concern targets the practical execution of blending standardized core sets with custom parameters. Critical voices caution that adding multiple product-specific outcomes on top of a standard COS is not a perfect compromise. Instead of preserving scientific freedom, this approach risks overloading the trial protocol, overcomplicating the data collection, and ultimately diluting or distorting the original focus of the research project:

*But if you say you can add additional parameters, use additional measurement instruments, that dilutes the study project in my view*.

[Interview 14, entrepreneur and consultant]

The second, more profound challenge lies in the current gap between methodological ideal and regulatory reality. For commercial digital health entrepreneurs, investing resources into alignment with a COS is only reasonable if it mitigates business risks. However, the data highlight deep uncertainty regarding whether compliance with a community-derived COS actually guarantees acceptance by regulatory bodies. As long as national authorities (such as the BfArM) do not officially endorse or enforce these specific core outcomes, manufacturers face the risk of designing a comparable study that still fails to achieve official market clearance or reimbursement approval:

*My question is rather, does it solve any problem for me as an entrepreneur, and I fear it doesn*’t. [...] *Because that’s the question again: What does the BfArM do? Because how do you want to establish the binding nature of these Core Outcomes?* [...] *What makes that the standard?*

[Interview 02, entrepreneur and consultant]

To bridge the gap between these opportunities and risks, the interviewees formulated concrete, nonnegotiable requirements that must be met for COS to achieve routine implementation in the research landscape.

The first requirement relates to fairness across indications. Participants stressed that the structural scope of COS must remain equitable and balanced across different therapeutic areas. If different medical fields develop core sets with heavily contrasting workloads—where manufacturers in one clinical indication only have to collect 3 outcomes, while competitors in another field are forced to track 5 or more—it would create market distortions. To achieve broad acceptance among developers, the procedural and financial burden of data collection must not result in an unfair disadvantage for specific treatment areas:

*At the latest, manufacturers would say, how can it be that some [*...*] let’s say only have to collect three points for their Core Set and others have to collect five points or so. So I imagine that as a challenge.*

*[Interview 03, physician *and *psychologist]*

The second requirement relates to pragmatic feasibility. Operational brevity is identified as a vital success factor. Because a COS is often envisioned as an additive framework rather than a total replacement for product-specific metrics, it must be kept as short as possible. In fast-paced digital health environments, the feasibility of assessment procedures depends on keeping the burden on both patients and study teams as low as possible. If a COS substantially increases the frequency or duration of assessments, its implementation may become impractical in routine clinical practice:

*And if it’s feasible, I mean Core Outcome Sets have to be short, because if everyone should do this and partly additionally, then it can’t multiply assessment times by five*.

[Interview 15, researcher]

## Discussion

### Principal Findings

Previous scientific literature has primarily focused on the effectiveness of individual DTx [46-49], cost-benefit or health economic evaluations [50-53], and factors facilitating or hindering their adoption and integration into clinical care [54-56]. As outlined in the *Introduction* section, previous studies have likewise emphasized the considerable heterogeneity in O&M used across DTx effectiveness studies [17,27-29]. However, little was known about how DTx studies are conceptually designed in practice and which methodological and content-related considerations guide this process. This knowledge gap was addressed in the present research. The results of this study provided an objectification of methodological approaches and content aspects of how O&M were selected in DTx studies, as well as a collection of suggestions for achieving better comparability of DTx studies and apps in the future.

In summary of the results addressing our first research question, we can conclude that the methodological approach for selecting O&M in DTx studies across all professional groups was primarily guided by research activity. Conducting an initial literature review during the early stages of study planning reflects established practice and aligns with the standards for developing high-quality clinical studies, even outside the DTx context [15,57-59]. However, when interpreting these findings, it should be noted that one inclusion criterion for participation in this study was prior DTx research activity. This criterion may have contributed to the prominence of research as a theme in the results.

The analysis of subcategories regarding the content aspects considered when selecting O&M revealed both commonalities and differing emphases. Overall, an imbalance in the total number of coded statements was observed. Within the group of interviewees classified as researchers, substantially more statements were coded compared with the other professional groups. It should also be noted that the professional groups differed in size. The physicians and psychologists group and the entrepreneurs and consultants group were based on 6 interviews each, whereas the researchers group was based on 7 interviews. On the basis of the present data, slight tendencies can be observed regarding which substantive aspects different professional groups emphasize when selecting O&M in DTx studies. However, these tendencies are not pronounced enough to support the conclusion that systematic, profession-related differences exist in the content aspects considered when selecting O&M.

In comparison with findings reported by other authors, the statements of our interview partners showed clear convergence. Ataiy et al [60] investigated the challenges faced by DiGA manufacturers when seeking approval of DTx within the German fast-track process. Their study participants identified the demonstration of a positive health care effect through scientific studies as the major challenge, with particular emphasis on the implementation of the study protocol, the recruitment of study participants, and the coordination of logistics. Similar challenges related to the practical implementation of planned study designs were also reported by our participants and were coded under the subcode Practical implementation and pragmatic aspects in the selection of O&M.

Furthermore, the statements of the interview partners aligned with the DTA’s emphasis on the use of clinically relevant and previously published end points in DTx studies [31]. However, contrary to the DTA’s recommendation to select patient-relevant end points whenever possible, such considerations were reported only rarely across the interview groups [31]. Especially within the physician and psychologist group, only 2 interviewees indicated that they consider aspects related to patients and target groups when selecting outcomes. Compared to other code categories, these statements were mentioned substantially less frequently. The limited incorporation of patient and end user perspectives into the development and evaluation of DTx has repeatedly been identified as a critical limitation in the existing literature [61,62]. Insufficient attention to co-design aspects can substantially limit the uptake and long-term use of DTx in real-world settings. A lack of alignment with users’ needs and everyday contexts may undermine sustained engagement [61,63-65]. Possible approaches for involving end users in the development and evaluation of DTx include focus group interviews, think-aloud strategies, surveys, and persona building, in which a persona represents a key end user [66].

The third section of the interviews aimed to identify suitable solution approaches for improving the comparability of DTx studies and apps. As pointed out by McLeod et al [67], the selection of appropriate end points for clinical trials of health care interventions has become increasingly challenging due to the growing diversity of study designs and the differing interests of end user groups. In their view, there is a lack of guidance for selecting suitable end points in clinical trials. Similar to these views, the most frequently expressed response by our interview participants was the need to develop guidance systems for O&M selection in DTx studies to improve comparability. This appears well suited as it is independent of country-specific legal requirements and enables international cooperation.

A similar approach is reflected in the interviewees’ suggestion to develop a registry or AI system to support the selection of O&M for DTx studies. While stricter legal frameworks and regulations must be understood as static concepts, a registry documenting which types of O&M have been used in other DTx studies, or an AI system instead provides a space and opportunity for exchanging ideas and approaches to the design of DTx studies among study investigators. In particular, the concept of an interactive AI reflects the principles of agile software research. The development and evaluation of a DTx software app cannot be divided into independent phases. Rather, they mutually influence each other and form a cyclical process [18,26]. An interactive AI generates a response to a user query while simultaneously allowing the user to engage with that response by suggesting modifications or corrections. From this perspective, both proposals represent feasible and practice-oriented solution concepts. To our knowledge, there is currently no open-access registry or AI system specifically designed for DTx studies that provides study information on investigated medical conditions and applied outcomes and measurements.

Nevertheless, the participants’ calls for more uniform international standards for development and approval, as well as their demands for more comprehensive and detailed reporting of DTx studies, were consistent with the statements of other authors [29,68,69]. For example, Seo et al [29] emphasized the need for standardized research designs and consistent methodological approaches to minimize confounding factors and enable clearer conclusions regarding the effectiveness of DTx across different disease areas. Similarly, Yao et al [68] highlighted that standardized and comprehensive reporting improves the reliability of study results and helps regulators evaluate interventions while protecting patient safety and rights. As outlined in the *Introduction* section, a solution frequently proposed in the literature to achieve these goals is the use of COS specifically designed for DTx studies.

According to the assessments of our interview participants, a major advantage of COS would lie in their potential to simplify research workflows and accelerate study planning. Previous work by Clayton et al [58] showed that the considerable time investment required often discourages researchers from conducting more comprehensive literature searches during study planning. As outlined in the literature and anticipated by our interview participants, COS could serve as a valuable tool to both ensure a minimum standard of study quality and support researchers in the planning process, including the identification of appropriate O&M [70,71]. Furthermore, the use of COS enables more efficient aggregation of study results in subsequent meta-analyses [41,71,72].

However, the results of our study provide a critically evaluative perspective on the use of COS in DTx studies, which has not yet been described in the literature. Our interview participants emphasized that COS should maintain an appropriate scope to allow their additional integration into study designs without placing an excessive burden on researchers or participants. They also highlighted the importance of regulatory acceptance and noted that COS need to be recognized by the authorities responsible for DTx approval. If such acceptance cannot be guaranteed, COS may still offer a theoretically valuable tool for improving comparability and quality across DTx studies, but they would not resolve an important challenge faced by study authors, namely the ongoing uncertainty about whether their DTx approval studies will be accepted by regulatory bodies. When interpreting these statements, it should be considered that they primarily reflect the perspective of study leads who have a direct interest in the acceptance and approval of their own DTx apps and study results. To obtain a more comprehensive understanding, future research should therefore examine the use of COS from the perspective of regulatory authorities to incorporate and assess their views as well.

To date, to the best of our knowledge, no COS have been specifically developed for DTx. An unanswered question is who takes on or coordinates this task. As noted by 1 interview participant, it can be hypothetically assumed that content interests regarding which O&M are considered relevant, and the level of detail required for corresponding COS, vary by stakeholder group. To avoid overrepresentation of individual interests, actors from all disciplines, including medical societies, researchers, insurance companies, politicians, providers, and patient representatives, should be involved in the development of COS [67,73,74].

### Limitations

Several limitations should be considered when interpreting the findings of this study. First, the results are based on a small cohort of interviewees, most of whom had experience conducting DTx studies within the DiGA authorization context in Germany. While this focus provided valuable insights due to Germany’s highly structured DTx authorization process, perspectives from study authors outside Germany were underrepresented.

Second, prior experience in conducting DTx studies was an inclusion criterion for participation. This may have introduced selection bias, as individuals actively engaged in research may be overrepresented. In addition, not all professional groups involved in DTx development, authorization, and financing could be included.

### Conclusions

The scientific contribution of this study lies in its detailed examination of the methodological approaches underlying the design of DTx studies, as well as the key conceptual aspects considered during their development. According to our findings, the methodological approach to selecting O&M in studies designed to demonstrate the clinical efficacy of DTx in the form of apps was likewise primarily based on research, as well as on personal prior experience and individual ideas. When selecting O&M, study authors particularly emphasized scientific quality criteria, alongside considerations of practical implementation and feasibility.

In addition, we explored the hypothesis frequently discussed in the literature that the use of COS may reduce heterogeneity in outcomes and measurement instruments and improve the comparability of DTx studies and apps. By directly interviewing authors of DTx studies, we assessed whether COS are perceived as a feasible and appropriate solution from the perspective of those actively involved in DTx research. Further research is required to clarify how COS for DTx can be developed, disseminated, and officially recognized depending on the therapeutic indication area.

Beyond evaluating COS as a proposed solution, our findings also point to additional potential approaches for addressing heterogeneity, including the use of AI-assisted systems, standardized outcome measures and instruments, and other methodological guidance frameworks.

Future DTx development requires a balance between research freedom, provider interests, and clear, uniform international authorization standards to ensure therapeutic quality and comparability [55,75]. Excessive regulatory hurdles may hinder innovation, causing research and development to shift to countries with lower requirements or be discontinued, while overly low standards risk authorizing DTx without sufficient efficacy or relevant O&M, misleading users and generating unnecessary costs for health insurance companies and end users. COS development does not end with publication. Ongoing reevaluation and adaptation are needed to maintain quality and reflect technological and medical progress [74].

## Supplementary material

10.2196/86437 Multimedia Appendix 1 Semistructured interview guide.

10.2196/86437 Multimedia Appendix 2 Primary coding system (details of the primary coding framework used in the qualitative analysis).

10.2196/86437 Multimedia Appendix 3 Secondary coding system—comprehensive secondary coding system with definitions and text examples.

10.2196/86437 Multimedia Appendix 4 Participant characterization—table showing demographic and professional details of the interview participants.

10.2196/86437 Multimedia Appendix 5 Distribution of subcode categories across interview groups.

10.2196/86437 Checklist 1 COREQ checklist.

## Abbreviations

BfArM: Bundesinstitut für Arzneimittel und Medizinprodukte
COREQ: Consolidated Criteria for Reporting Qualitative Research
COS: core outcome sets
DiGA: digital health applications or Digitale Gesundheitsanwendung (German)
DTA: Digital Therapeutics Alliance
DTx: digital therapeutics
INAMI-RIZIV: National Institute for Health and Disability Insurance
MCID: minimal clinically important difference
O&M: outcome and measurement methods
