# Supplementary material for: Selection of Outcomes and Measurement Methods in Clinical Trials for Digital Therapeutics: Qualitative Analysis
Source: JMIR Form Res. 2026 Oct 15;10:e86437. doi: 10.2196/86437 (PMC13671066; doi:10.2196/86437)
Supplement: Supplementary file 1 [file formative-v10-e86437-s001.docx]

**Guideline-based qualitative expert interviews as part of the study**

"Theory and practice - possibilities and ways to evaluate the evidence of digital health applications"

# Introduction

Thank you very much for agreeing to be available to us as an interview partner as part of the

study. To begin with, I would like to briefly introduce myself and give you an overview of our

research topic.

**Introduction of the interviewer**

Name, chair, academic and professional background

**Introduction of the research topic:**

In this study, we would like to investigate the procedures for selecting outcomes and

measurement methods in studies on digital therapeutics (DTx) in the specific form of apps.

Example: A new app has been developed for pain therapy for back pain. We want to test the

effectiveness of the application in a study. How do we go about selecting what we want to

measure in our study (outcome) and how we want to measure it (measurement method)?

**Procedure of the interview**

Over the next 30-45 minutes, I would like to talk to you about three topics using a semistructured interview guide:

1) Getting to know the interviewee

2) Your approach & thoughts on what should be considered when selecting measurement

methods and outcomes in Digital Therapeutics (DTx) studies

3) Your recommendations for future studies on digital therapeutics (DTx)

**General & Legal**

The important thing is that there is no right or wrong answer. We are more interested in

what you think about the topics mentioned.

You have the option of cancelling the interview at any time during the interview without

giving reasons. This will not put you at a disadvantage. In this case, your participation data

will be deleted immediately and will not be included in the study.

Prior to the interview, you have already received the information letter with information on

anonymity, processing and protection of your data and have given your written consent to

this.

In order to enable the subsequent evaluation of the interview, I would like to record it in accordance with the information provided in the information sheet. Do you agree to this?

Do you have any unanswered questions?

**Information:** The audio recording will start now.

# 1) Getting to know the interviewee

- May I ask you how old you are (in years).
- Which gender would you categorise yourself as?
- Please describe in one sentence what your current job is.
- How many years of work experience do you have (total, in years)?
- How many studies on DTx have you participated in so far (not a test person)?
- Where were the majority of the studies in which you were involved conducted?

☐ Domestic (Germany)

☐ Abroad (not Germany)

☐ Equal ratio between home and abroad

- Please categorise your experience in planning and conducting studies on Digital

Therapeutics (DTx) into a speciality / subject area / clinical picture (e.g. cardiology, gynaecology)

# 2) Your recommendations on what should be considered when selecting outcomes and measurement methods in studies on digital therapeutics (DTx).

The following section deals with outcomes and measurement methods in DTx studies and

the question of how to select them for your studies and what to use as a guide. If you use

guidelines / standards for orientation, please name them specifically. If you have gained

personal experience (that was particularly good / bad), please let me know.

We start with ...

- Selection of **outcomes** (e.g. pain intensity or quality of life)
  - How do you proceed with the selection?
  - What do you pay attention to?
  - Do you use guidelines / standards to guide the selection of outcomes?
    - Please name them.
- Selection of **measurement methods** (numeric rating scale, Generalised Anxiety Disorder-7 (GAD-7))
  - How do you proceed with the selection?
  - What do you pay attention to?
  - Do you use guidelines / standards to guide the selection of outcomes?
    - Please name them.

# 3) Your recommendations for future studies

- Name **ways** to increase the comparability of studies on digital therapeutics (DTx) in the future.
- If uniform …
  - **Outcomes** in studies on digital therapeutics (DTx)
    - Advantages?
    - Disadvantages?
    - Which predominates (please give reasons)?
  - **Measurement methods** in studies on digital therapeutics (DTx)
    - Advantages?
    - Disadvantages?
    - Which predominates (please give reasons)?
- Your opinion on our example proposal:

Use of **core outcome sets in studies to demonstrate the medical benefit of DTx.** Standardised outcomes and measurement methods are used in relation to a clinical picture / topic area (e.g. pain reduction). These are regarded as minimum requirements; further studies are possible.

Please answer yes/no I think it is (not) good and give reasons for your answer:

☐ Yes I think it is goog

☐ No I do not think it is good

Justification:

# End of the interview

We have now reached the end of our interview.

Before we conclude the interview - are there any open points or questions from your side?

How understandable were the interview questions for you? (0=very incomprehensible, 5=completely comprehensible)

Information: The audio recording ends now.

Do you have any suggestions or feedback?

I would like to take this opportunity to thank you for your participation and say goodbye.
