# Supplementary material for: Selection of Outcomes and Measurement Methods in Clinical Trials for Digital Therapeutics: Qualitative Analysis
Source: JMIR Form Res. 2026 Oct 15;10:e86437. doi: 10.2196/86437 (PMC13671066; doi:10.2196/86437)
Supplement: Supplementary file 2 [file formative-v10-e86437-s002.docx]

**Multimedia Appendix 2- Primary coding system** *[Details of the primary coding framework used in the qualitative analysis]*

- Methodological Approach

➤ Outcomes

➤ Measurement Methods

- Content Aspects

➤ Outcomes

➤ Measurement Methods

- Proposals for Improving Comparability
- Core Outcome Set Application

➤ COS Advantages

➤ COS Disadvantages
