# Supplementary material for: Selection of Outcomes and Measurement Methods in Clinical Trials for Digital Therapeutics: Qualitative Analysis
Source: JMIR Form Res. 2026 Oct 15;10:e86437. doi: 10.2196/86437 (PMC13671066; doi:10.2196/86437)
Supplement: Supplementary file 3 [file formative-v10-e86437-s004.docx]

**Multimedia Appendix 4:** Participant characterization - table showing demographic and professional details of the interview participants.

| **Document Name** | **Gender** | **Age** | **Professional Group** | **Years of Work Experience** | **Number of Studies Conducted with DTx Reference** | **Country Where Most DTx-Related Studies Were Conducted** |
| --- | --- | --- | --- | --- | --- | --- |
| Interview_01 | F | 36 | Researchers | 11 | 14 | Germany |
| Interview_04 | F | 47 | Researchers | 27 | 4 | Germany |
| Interview_09 | M | 38 | Researchers | 10 | 4 | Abroad (Switzerland) |
| Interview_11 | F | 35 | Researchers | 12 | 1 | Germany |
| Interview_12 | F | 28 | Researchers | 3 | 5 | Germany |
| Interview_13 | F | 27 | Researchers | 3 | 4 | Germany |
| Interview_15 | M | 50 | Researchers | 20 | 7 | Germany |
| Interview_03 | M | 31 | Physicians & Psychologists | 6 | 5 | Germany |
| Interview_05 | M | 60 | Physicians & Psychologists | 32 | 10 | Abroad (Switzerland) |
| Interview_06 | M | 42 | Physicians & Psychologists | 11 | 4 | Germany |
| Interview_08 | M | 43 | Physicians & Psychologists | 17 | 20 | Abroad (Switzerland) |
| Interview_16 | M | 41 | Physicians & Psychologists | 13 | 3 | Germany |
| Interview_17 | M | 36 | Physicians & Psychologists | 5 | 6 | Germany |
| Interview_02 | M | 49 | Entrepreneurs & Consultants | 20 | 1 | Germany |
| Interview_07_1 | M | 48 | Entrepreneurs & Consultants | 25 | 15 | Germany |
| Interview_07_2 | F | 45 | Entrepreneurs & Consultants | 21 | 10 | Germany |
| Interview_10 | M | 36 | Entrepreneurs & Consultants | 8 | 5 | Germany |
| Interview_14 | F | 51 | Entrepreneurs & Consultants | 25 | 6 | Germany |
| Interview_18 | M | 72 | Entrepreneurs & Consultants | 45 | 3 | Germany |
